# Epithelial-to-Mesenchymal Transition in Pancreatic Cancer is associated with Restricted Water Diffusion in Diffusion-Weighted Magnetic Resonance Imaging

**DOI:** 10.7150/jca.63633

**Published:** 2021-11-04

**Authors:** Philipp Mayer, Anne Kraft, Wilfried Roth, Thilo Hackert, Frank Bergmann, Hans-Ulrich Kauczor, Verena Steinle, Ekaterina Khristenko, Miriam Klauss, Matthias M. Gaida

**Affiliations:** 1 Clinic for Diagnostic and Interventional Radiology, Heidelberg University Hospital, 69120 Heidelberg, Germany.; 2 Institute of Pathology, University Medical Center Mainz, JGU-Mainz, 55131 Mainz, Germany.; 3 Department of General, Visceral, and Transplantation Surgery, Heidelberg University Hospital, 69120 Heidelberg, Germany.; 4 Institute of Pathology, Heidelberg University Hospital, 69120 Heidelberg, Germany.; 5 Clinical Pathology, Klinikum Darmstadt GmbH, 64283 Darmstadt, Germany.; 6 Research Center for Immunotherapy, University Medical Center Mainz, JGU-Mainz, 55131 Mainz, Germany.; 7 Joint Unit Immunopathology, Institute of Pathology, University Medical Center, JGU-Mainz and TRON, Translational Oncology at the University Medical Center, JGU-Mainz, 55131 Mainz, Germany.

**Keywords:** Pancreatic cancer, epithelial-to-mesenchymal transition, diffusion-weighted magnetic resonance imaging.

## Abstract

**Purpose:** This study aimed to evaluate the potential of diffusion-weighted magnetic resonance imaging (DW-MRI) as imaging biomarker for epithelial-to-mesenchymal transition (EMT) in pancreatic ductal adenocarcinoma (PDAC).

**Methods:** In forty-two patients, preoperative apparent diffusion coefficient (ADC) values of therapy-naive PDAC were compared with immunohistochemical expression profiles of the epithelial marker E-cadherin as well as mesenchymal transcription factors Runt-related transcription factor 2 (Runx2) and Zinc finger E-box-binding homeobox 1 (Zeb1), as determined by Allred immunoreactivity score.

**Results:** We observed a significant positive rank correlation between the ADC and the E-cadherin Allred score (ρ = 0.553, p < 0.001) and significant negative rank correlations between the ADC and the Runx2 Allred score (ρ = -0.526, p < 0.001) as well as the Zeb1 Allred score (ρ = -0.710, p < 0.001). Compared to tumors with low ADC values < 1.3 µm^2^/s, tumors with ADC values ≥ 1.3 µm^2^/s had significantly higher Allred scores for E-cadherin (median, 4 *versus* 5; p < 0.001) and significantly lower Allred scores for Runx2 (median, 3 *versus* 2; p = 0.003) as well as Zeb1 (median, 4 *versus* 0; p < 0.001).

**Conclusion:** In PDAC, tumor plasticity in terms of EMT is well reflected by ADC values from DW-MRI. In the near future, DW-MRI could be beneficial for identification of PDAC patients that might profit from personalized EMT-targeted therapies.

## Introduction

Pancreatic ductal adenocarcinoma (PDAC) is a highly aggressive tumor, which is predicted to achieve the second rank in cancer-associated deaths in 2030 1. Since most early-stage tumors are asymptomatic, the majority of PDACs are detected at a progressed stage and only a minor portion of patients qualify for potentially curative resection 2. Other factors contributing to the poor prognosis of PDAC include early vascular invasion, the rapid development of distant metastases, and its notoriously poor response to (radio)chemotherapy 3.

Clinical imaging plays a key role not only for the initial diagnosis of PDAC, but also for the assessment of the local tumor mass, detection of distant metastases, and monitoring of disease in patients undergoing (neo-)adjuvant chemotherapy as well 4. Computed tomography (CT) is the most widely used imaging modality to assess the local tumor spread and particularly the involvement of major abdominal vessels which determines tumor resectability 5,6 as well as extrapancreatic perineural invasion which is a significant predictor of recurrence 7. A couple of studies reported conventional contrast-enhanced CT to be useful for characterization of some microstructural features of PDAC, such as cellular density 8 and stroma content 9. However, the major strength of conventional contrast-enhanced CT is rather the accurate depiction of the macroanatomy than the probing of the tissue microstructure 5.

Magnetic resonance imaging (MRI) is often seen as a problem-solving tool for detection and characterization of PDACs 10. Its superior soft tissue contrast is beneficial for detection of small non-contour deforming tumors, and for detection of a minor portion of PDACs that are isoattenuating on CT without any visible tumor-pancreas contrast 10,11. In particular, diffusion-weighted (DW)-MRI, which quantitates the random motion of water molecules in biological tissues, has a very high diagnostic performance for detection of PDACs with reported sensitivity and specificity values well above 90% 12. In recent years, several studies have reported encouraging results for (sub)cellular characterization of PDAC lesions by DW-MRI 12, including their cell density 13, growth pattern 14, stromal content 15, immune cell infiltration 16, as well as tumor hypoxia 17. The ultrastructural findings in PDAC show a heterogeneous picture with tumor cells sealed by an extensive fibrotic stroma with interspersed inflammatory cells 18. PDAC shows usually a glandular growth pattern of tumor cells, however also (micro-) papillary, solid-nest-like, cribriform, or single-cell dissociated tumor growth can be detected, reflecting the high plasticity of this cancer 14. Various molecular factors, such as the loss of epithelial markers and the gain of a mesenchymal tumor phenotype (epithelial-to mesenchymal-transition; EMT) or dense infiltrates of neutrophils, are associated with changes in the microarchitecture and tumor growth pattern 19,20. In PDAC, as in a variety of other tumors, EMT is associated with an aggressive, invasive phenotype, where tumor cells disseminate from the main tumor mass and change their morphology and molecular phenotype, towards anti-apoptotic, pro-metastatic, and chemoresistant behavior 21. The process of EMT in tumor cells can be induced by a variety of environmental factors, for example numerous cytokines, such as transforming growth factor (TGF)-β, by loss of cell-cell contact, by proteases, matrix stiffness, or by tumor hypoxia 20,22-24. Typical features of EMT besides the change of cell morphology and loss of polarity, are the downregulation of the epithelial cell adhesion molecule E-cadherin and different cytokeratins, in parallel to the upregulation of mesenchymal markers such as vimentin or fibronectin 22. This process is induced by transcription factors such as the Runt-related transcription factor 2 (Runx2), as an early regulator of EMT, or the Zinc finger E-box-binding homeobox 1 (Zeb1), as a later occurring key EMT regulator 25,26. In PDAC undergoing EMT, microarchitectural changes are described, such as the separation of tumor cells from the main tumor mass and disseminated tumor growth in small clusters or as single cells, but also the loss of cytokeratins and expression of EMT-promoting transcription factors Zeb1 or nuclear β-Catenin 19,20,27. Of note is that EMT can confer resistance to radiochemotherapy. Targeted inhibition of Zeb1 is known to increase radiosensitivity of cancer cells through an EMT-dependent mechanism 28. In a study by Arumugam et al., high levels of Zeb1 and low levels of E-cadherin in PDAC cell lines were correlated with resistance to gemcitabine, 5-fluorouracil (5-FU), and cisplatin 29.

Several strategies targeting EMT in PDAC are being investigated in preclinical studies 30 and targeting EMT could increase PDAC's sensitivity to conventional radiochemotherapy in the future.

To date, there are no clinically established imaging biomarkers for prediction of EMT *in vivo*. While contrast-enhanced CT is widely used to assess the macroscopic tumor extent, DW-MRI can probe the tissue composition at a more granular scale 12. DW-MRI was proven to well reflect tissue properties of PDAC that are closely linked to EMT, including tumor growth pattern 14, stroma content and properties 15, as well as tumor hypoxia 17, and previous studies reported an association of DW-MRI and EMT in xenograft tumor models 31,32. Therefore, we hypothesized that DW-MRI could represent a biomarker for EMT in PDAC.

## Material and Methods

### Patients

**Figure 1:**
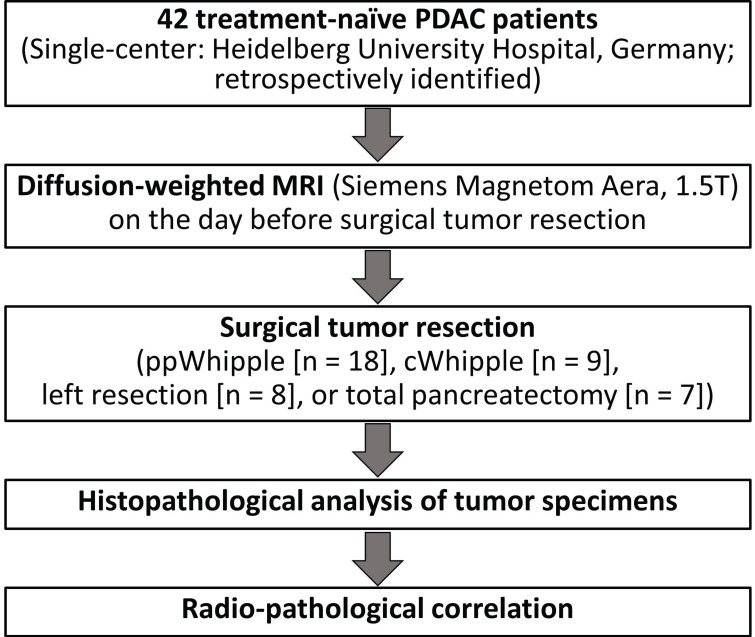
Workflow of the study.

The study protocol was approved by the local Ethics Committee (S-044/2012). Forty two PDAC patients from a previously well-characterized cohort 17 were retrospectively included in the present study. All patients had surgical resection of therapy-naive PDAC and had a DW-MRI of the pancreas on the day before surgical tumor resection at the University Hospital of Heidelberg, Germany. Written informed consent was obtained from all participants in the study. The detailed clinical parameters are listed in Table 1. The workflow of the study is presented in Figure 1.

### Diffusion-weighted MRI

DW-MRI was performed on a clinical 1.5 Tesla scanner (Magnetom Aera, Siemens Healthcare, Erlangen, Germany) using a 6-element body-phased array coil and a 24-channel spine array coil. DW-sequence was a single-shot echo-planar imaging sequence in axial orientation. Acquisition parameters were as follows: Number of slices per b-value, 14; slice thickness, 5.0 mm; distance factor, 5 %; phase encoding direction, anterior-posterior; repetition time (TR), 2200 ms; time to echo (TE), 58,0 ms; acquisition matrix, 130 * 92; spectral fat saturation; bandwidth, 2262 Hz/pixel; GRAPPA (generalized autocalibrating partially parallel acquisitions) acceleration factor, 2. All DW-MR examinations included at least the b-values b_0_ = 0 s/mm^2^, b_50_ = 50 s/mm^2^, and b_800_ = 800 s/mm^2^. Opposed to our previous study where apparent diffusion coefficients had been calculated from b_50_ and b_800_ (ADC_50,800_) 17, ADC values were now calculated from b_0_ and b_800_ (ADC_0,800_; in the following referred to as ADC).

The MITK (Medical Imaging Interaction Toolkit) diffusion application (version 2018.09.99), which had been developed by the Division of Computer-assisted Medical Interventions and the Division of Medical Image Computing of the German Cancer Research Center (DKFZ), was used for DW-MRI analysis 33. Two expert pancreatic radiologists independently drew three-dimensional volumes of interest (VOIs) surrounding the tumors on DW-images. Corresponding anatomic MR images and/ or CT images were available for every patient to help determine the exact anatomical outlines of the tumors. ADC values of tumors were then calculated from b_0_ and b_800_ using MITK diffusion.

### Immunohistochemistry

Human tissue samples were provided by the local tissue bank in agreement with the regulations of the tissue bank and the approval of the local Ethics committee (no. 206/2005). All patients gave written informed consent. Paraffin sections of the respective tumor specimens were used for immunohistochemistry, which was performed at the Institute of Pathology, University Medical Center Mainz, Germany. Prior to incubation with the primary antibody, heat-induced antigen retrieval was conducted using buffer (pH 6.0 for Runx2; pH 9.0 for E-cadherin and Zeb1; Dako EnVision, Glostrup, Denmark). The following primary antibodies were used: E-cadherin (ready to use; Dako), Runx2 (1:50; Cell Signaling Technology, Leiden, Netherlands), Zeb1 (1:500; Novus Biological, Abingdon, UK).

Antibody-binding was visualized with DAB+ chromogen (Dako) and analyzed using the semi-quantitative and widely established Allred scoring system, the sum of staining intensity and distribution. The expected staining of E-cadherin is membranous, of Runx2 and Zeb1 nuclear. The analyses were performed on whole tumor sections to overcome heterogeneity of PDAC. Images were taken using the Gryphax Subra camera system (Jenoptik, Jena, Germany). For confirmation of results, quantification was cross-checked software-based by ImageJ (US National Institutes of Health, Bethesda, MD, USA).

### Statistical analyses

Statistical analyses were performed using the statistical software package MedCalc (version 20, MedCalc Software Ltd, Ostend, Belgium). Data were expressed as absolute and relative frequencies for categorical data and as median with interquartile range (IQR) for continuous variables. The Mann-Whitney U test was used for comparison of continuous variables between groups. Correlation analyses were performed using Spearman's rank correlation coefficient. Inter-rater reliability was assessed with the intra-class correlation coefficient (ICC) (two-way ICC, random raters' assumption reproducibility). As proposed by Song et al. 34, ICC values were interpreted as poor correlation (0.00 - 0.20), fair correlation (0.21 - 0.40), moderate correlation (0.41 - 0.60), good correlation (0.61 - 0.80), or excellent correlation (0.81 - 1.00). P < 0.05 were considered to be statistically significant.

## Results

### Diffusion-weighted imaging analyses

Tumor ADC values could be determined for all patients. Agreement for ADC values between both raters was excellent with ICC = 0.856 (95% confidence interval (CI), 0.748 - 0.920). In the following, given ADC values represent mean values for both raters, if not stated otherwise. ADC values of tumors ranged from 0.959 µm^2^/s to 1.631 µm^2^/s (median, 1.297 µm^2^/s; IQR, 1.166 - 1.419 µm^2^/s) and were similar to those from previous studies on DW-MRI in treatment naïve PDAC patients 35-38.

### Immunohistochemical analyses

E-cadherin showed a membranous staining pattern. E-cadherin Allred scores ranged from 0 to 8 (median, 5; IQR, 4 - 6). Runx2 and Zeb1 showed a nuclear staining pattern with Allred scores from 0 to 7 for Runx2 (median, 2; IQR, 2 - 4) and from 0 to 5 for Zeb1 (median, 2.5; IQR, 0 - 4). There was a significant negative correlation between the E-cadherin Allred score and the Zeb1 Allred score (ρ = -0.604, p < 0.001) and a significant positive correlation between the Runx2 Allred score and the Zeb1 Allred score (ρ = 0.507, p < 0.001). The E-cadherin Allred score was only weakly negatively correlated with the Runx2 Allred score (ρ = -0.278, p = 0.074). E-cadherin, Runx2, and Zeb1 Allred scores were neither significantly different between T2 tumors and T3 tumors (p ≥ 0.294) nor between moderately differentiated (G2) and poorly differentiated tumors (G3, p ≥ 0.422) or between lymph node negative (N0) and positive tumors (N1 or N2, p ≥ 0.704).

### Radiopathological correlation

**Figure 2:**
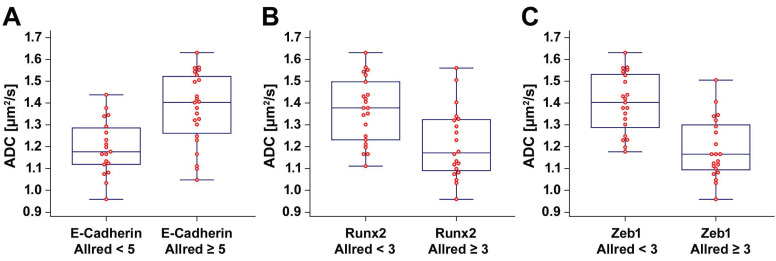
** Box-and-whisker plots.** The box-and-whisker plots show the distribution of ADC values relative to Allred scores of E-cadherin (A), Runx2 (B), and Zeb1 (C).

We observed a significant positive correlation between the ADC and the E-cadherin Allred score (ρ = 0.553, p < 0.001). There were significant negative correlations between the ADC and the Runx2 Allred score (ρ = -0.526, p < 0.001) as well as between the ADC and the Zeb1 Allred score (ρ = -0.710, p < 0.001). ADC values were significantly lower in tumors with low E-cadherin expression (E-cadherin Allred < 5; median ADC, 1.170 µm^2^/s; IQR, 1.119 - 1.286 µm^2^/s) than in tumors with high E-cadherin expression (E-cadherin Allred ≥ 5; median ADC, 1.403 µm^2^/s; IQR, 1.261 - 1.522; p = 0.001) (Figure 2A). ADC values were significantly higher in tumors with Runx2 Allred scores < 3 (median ADC, 1.378 µm^2^/s; IQR, 1.231 - 1.498 µm^2^/s) than in tumors with Runx2 Allred scores ≥ 3 (median ADC, 1.172 µm^2^/s; IQR, 1.090 - 1.324 µm^2^/s; p = 0.003) (Figure 2B). Tumors with Zeb1 Allred scores < 3 had significantly higher ADC values (median ADC, 1.403 µm^2^/s; IQR, 1.288 - 1.532 µm^2^/s) than tumors with Zeb1 Allred scores ≥ 3 (median ADC, 1.166 µm^2^/s; IQR, 1.094 - 1.300 µm^2^/s; p < 0.001) (Figure 2C). Vice versa, compared to tumors with ADC values < 1.3 µm^2^/s, tumors with ADC values ≥ 1.3 µm^2^/s had significantly higher Allred scores for E-cadherin (median Allred, 4; IQR, 3 - 5; *versus* median Allred, 5; IQR, 5 - 7.25; p < 0.001) and significantly lower Allred scores for Runx2 (median Allred, 3; IQR 2 - 4; *versus* median Allred, 2; IQR, 0 - 3; p = 0.003) as well as Zeb1 (median Allred, 4; IQR, 2.75 - 4.25; *versus* median Allred, 0; IQR, 0 - 2.25; p < 0.001).

ADC values were neither significantly different between T2 tumors and T3 tumors (p = 0.950) nor between moderately differentiated (G2) and poorly differentiated tumors (G3, p = 0.654) or between lymph node negative (N0) and positive tumors (N1 or N2, p = 0.353). This is in line with the results from a study by Rosenkrantz et al. 39 but in contradiction to the results from a study by Wang et al. 40.

**Figure 3:**
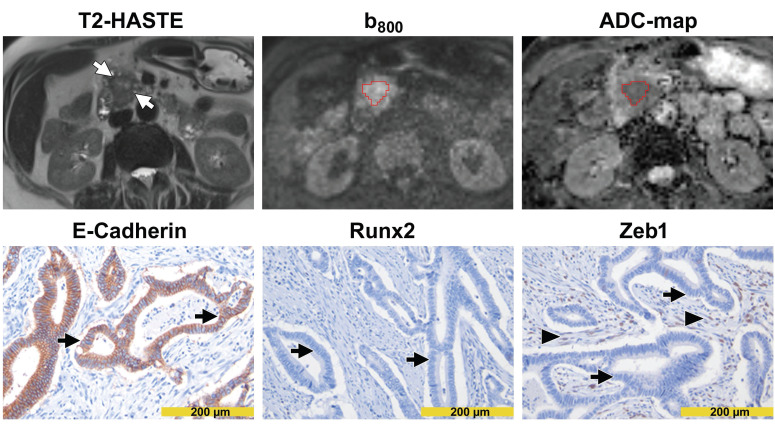
** Images from a patient with high diffusivity.** The T2-HASTE (Half-Fourier-Acquired Single-shot Turbo spin Echo) MR image shows the tumor in the pancreatic head (*arrows*). A VOI in red encompasses the tumor on the DW-MR images. Please note that the tumor is moderately hyperintense in the b_800_ image and moderately hypointense in the ADC map. The ADC value of the tumor was 1.560 µm^2^/s. In immunohistochemistry (*lower row*), tumor cells showed a diffuse and strong E-cadherin expression (*arrows in the left image*) but were widely negative for Runx2 (*arrows in the middle image*) and Zeb1 (*arrows in the right image*). Please note the Zeb1-positive stroma cells (*arrowheads in the right image*).

**Figure 4:**
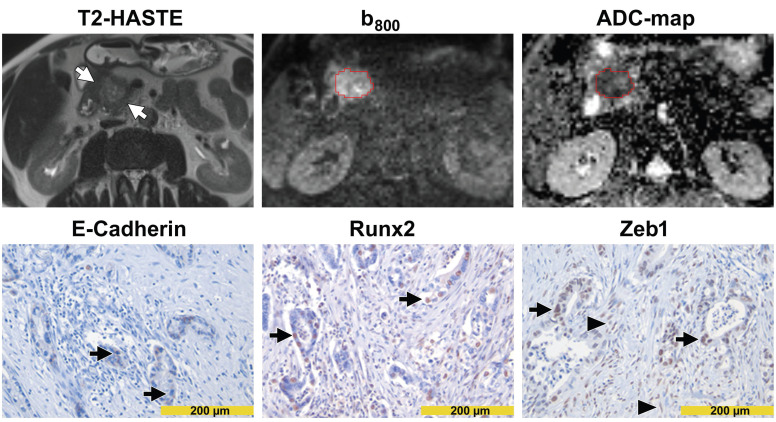
** Images from a patient with low diffusivity.** The T2-HASTE MR image shows the tumor in the pancreatic head (*arrows*). A VOI in red encompasses the tumor on the DW-MR images. Please note that the tumor is markedly hyperintense in the b_800_ image and markedly hypointense in the ADC map. The ADC value of the tumor was 1.048 µm^2^/s. In immunohistochemistry (*lower row*), tumor cells showed only focal membranous E-cadherin expression (*arrows in the left image*) but showed strong nuclear positivity for Runx2 (*arrows in the middle image*) and Zeb1 (*arrows in the right image*). Please note the Zeb1-positive stroma cells (*arrowheads in the right image*).

**Figure 5:**
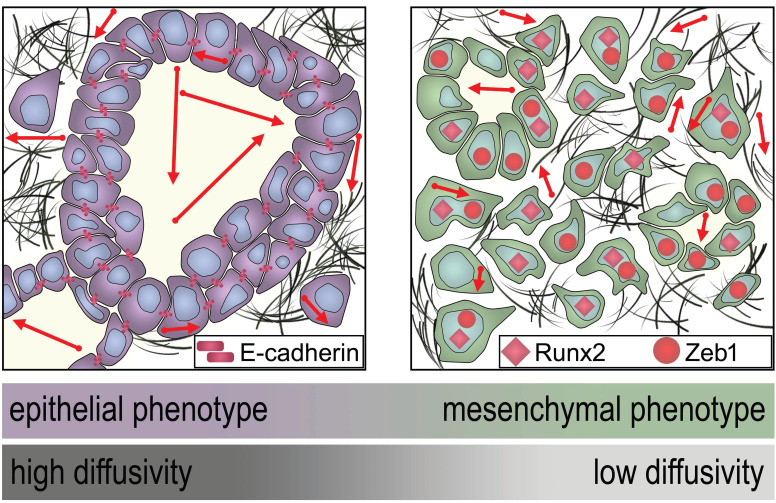
Schematic representation of the dependency of the MRI diffusivity on EMT. Epithelial type PDAC with high expression of E-cadherin exhibit significantly higher diffusivity (arrows) in DW-MRI than mesenchymal type PDAC with low expression of E-cadherin and higher expression of Runx2 and Zeb1.

DW images and immunohistochemical stainings from a patient with high diffusivity and epithelial tumor phenotype are presented in Figure 3. DW images and immunohistochemical stainings from a patient with low diffusivity and mesenchymal tumor phenotype are presented in Figure 4. A schematic representation of the dependency of the diffusivity on EMT in PDAC is presented in Figure 5.

## Discussion

Pancreatic cancer is characterized by high tumor plasticity, which is responsible for its aggressive behavior and therapeutic resistance 3,41. In this vein, multiple studies described molecular features being associated with this particular tumor aggressiveness. A well-established concept is epithelial to mesenchymal transition in PDAC, where tumor cells lose their epithelial features and de-differentiate into a mesenchymal, aggressive cellular phenotype, which is characterized by anti-apoptotic, pro-invasive, pro-metastasic, and chemoresistant features 21. EMT in pancreatic cancer can be visualized by disseminated tumor growth, loss of cytokeratins, or expression of the mesenchymal transcription factors Zeb1 or nuclear β-Catenin 19,20,27. We decided to use the established epithelial marker E-cadherin, and two mesenchymal markers, Runx2 as early-stage marker for EMT, and Zeb1 as later occurring marker. Epithelial features could be shown in the majority of tumors and were negatively correlated with the expression of the mesenchymal markers Runx2 and Zeb1, indicating a transient and dynamic EMT process in the tissues. The inverse correlation of E-Cadherin and Zeb1 is well documented for EMT in pancreatic cancer, resulting in consequent resistance to the clinically widely used chemotherapies 29. In line with these findings, also the expression of the second investigated EMT marker, Runx2, in pancreatic cancer is associated with chemoresistance 42. To date, prediction of EMT and other molecular markers indicating EMT-mediated aggressiveness or chemoresistance of PDAC is only possible using a tissue biopsy, because of the lack of established imaging biomarkers *in vivo*. We hypothesized that DW-MRI, which is widely used for pancreatic imaging, could represent an imaging biomarker for EMT since it was previously proven to reflect histological tissue characteristics of PDAC which are closely linked to EMT, tumor growth pattern 14, immune cell infiltrates 14, desmoplastic stroma characteristics 15, and tumor hypoxia 17, although the direct prove of EMT and imaging markers have not been established in PDAC. In the present study, we found a significant positive correlation between the ADC and E-cadherin expression in tumor cells. On the other hand, the ADC was significantly negatively correlated with Runx2 as well as Zeb1 expression. ADC values were significantly lower in tumors with low E-cadherin expression compared to high E-cadherin expression. Supporting that notion of an association with intratumor EMT, the ADC values were significantly higher in tumors with high Allred immunoreactivity scores for Runx2 and Zeb1 than in tumors with low Runx2 and Zeb 1 expression. Vice versa, compared to tumors with low ADC values, tumors with high ADC values had significantly higher expression of E-cadherin and significantly lower expression of Runx2 as well as Zeb1. Our data clearly show that intratumor EMT is closely linked to restricted diffusion in MRI. A possible explanation is that the larger glandular lumina and stable glandular structures of epithelial phenotype PDAC enable a relatively unimpeded diffusion of water molecules while the mesenchymal phenotype PDAC exhibits a more clustered growth pattern, sometimes even micro- or non-glandular and single cellular 27 which had been linked to more restricted water diffusivity 14. In line with this, Zhang et al. also reported a positive, albeit weak, correlation between the epithelial marker E-cadherin and the ADC in a chronic pancreatitis rat model 43. Similarly, in a study on EMT in a colorectal carcinoma xenografts model, tumors with ongoing EMT had lower diffusivity values than tumors without EMT 32. In seeming contradiction, Chen et al. found less restricted diffusion in mesenchymal-like mouse xenograft tumors than in their epithelial-like counterparts, using hepatocellular carcinoma (HCC) cell lines 31. They hypothesized that the tighter cell-cell junctions in the epithelial-like tumors lead to more densely packed tumor cells with decreased extracellular space, and thus more restricted diffusion than in the mesenchymal-like tumors 31. From the histological side, this effect is explainable because injected xenograft models form a solid tumor mass, without a desmoplastic stroma or glandular tumor formation, as in human (pancreatic) cancer. With intact epithelial adhesion molecules, a solid tumor with stable cell-cell contacts, but without significant intercellular spaces is outgrown, compared to the mesenchymal xenograft, which due to loss of intercellular contacts, creates small spaces, where water molecules can diffuse freely. This effect is obviously less prominent in the human PDAC in the present study: Cancer areas with high expression of epithelial markers form glandular structures with large intercellular spaces, surrounded by desmoplastic stroma. Mesenchymal cells from small or single cellular clusters, without larger empty spaces, and are therefore sealed in desmoplastic stroma.

Since EMT induction can be associated with increased glucose uptake of tumor cells 44, several studies focused on ^18^F-fluorodeoxyglucose (^18^FDG) uptake in positron emission tomography (PET) as an imaging biomarker for EMT in different tumor types, including esophageal cancer 45,46, HCC 47, oral squamous cell carcinoma 48, and non-small cell lung cancer 44. However, high costs, the required logistics, and limited availability are obvious drawbacks of ^18^FDG-PET 49,50, potentially restraining its widespread clinical use. On the other hand, DW-MRI is not limited by these drawbacks of ^18^FDG-PET and might therefore represent a more practicable imaging biomarker for the prediction of intratumor EMT.

We provide first-time evidence for a human pancreatic cancer cohort that tumor plasticity in terms of EMT is directly correlated to ADC values in DW-MRI. Non-invasive estimation of EMT in PDAC by DW-MRI could help to identify aggressive PDAC lesions with high risk for chemotherapy failure. In the future, DW-MRI may also be a helpful tool to select patients for personalized (experimental) therapies, targeting EMT 30.

Progress in (personalized) treatment methods for PDAC is of urgent need due to the still devastating prognosis of the disease. While recent advances in personalized medicine have led to significant improvements in overall survival in other solid malignancies, there have been only modest survival improvements for PDAC patients 51. This failure cannot be attributed to a scarcity of research in personalized medicine in PDAC 51. While previously used targeted therapies were limited by an insufficient understanding of the molecular aspects of the disease, better preclinical models mimicking human disease have led to a rapid increase in knowledge on PDAC's genetics and biology 52. New therapeutic strategies include exploitation of DNA repair abnormalities, targeting cancer-cell metabolism and EMT as well as components of the stroma or the tumor micromilieu 52-54. However, although up to one fourth of PDAC harbour clinically actionable molecular alterations and molecularly matched therapies may improve overall survival in these patients, the percentage of PDAC patients who ultimately receive targeted therapies is very low 51,55,56. This shows that the challenge for personalized medicine in PDAC might not only be the dearth of targeted and other novel therapy options, but rather improving patient stratification and making novel therapies more accessible to individual patients and treating physicians 51,52. Sole reliance on molecular markers for treatment stratification can be problematic in PDAC since tiny biopsy specimens often do not contain enough tumor cells, depending on the exact biopsy technique and the abundance of the stroma, and their results are affected by the biopsy site due to the notorious tumor heterogeneity of PDAC 51. Radio-pathological correlation studies, such as the present study, lead to the understanding that many microscopic and molecular alterations of the tumor tissue are accompanied by inter- and intraindividual qualitative and quantitative changes in radiological imaging 57. Therefore, radiological imaging, especially quantitative imaging, which allows non-invasive analysis of the whole tumor tissue, in combination with pathological and molecular tissue analysis, could serve as a valuable vein for treatment stratification in the era of personalized medicine 51.

In summary, the present study is an example that microarchitectural changes, seen in histopathological correlates, are directly reflected by MRI. The clinical impact is an upfront estimation, whether the tumor belongs to an aggressive, chemoresistant phenotype, which can be generated in the initial tumor staging and where potentially in the future PDAC patients may benefit from EMT-targeted therapies.

## Figures and Tables

**Table 1:** Clinical and pathological patient characteristics

Median Age (IQR)	67.5 years (42 - 85 years)
**Sex** Female Male	19 (45.2 %) 23 (54.8 %)
**Neoadjuvant (radio)chemotherapy** No Yes	42 (100.0 %) 0 (0.0 %)
**T stage** T1 T2 T3 T4	0 (0.0 %) 19 (45.2 %) 23 (54.8 %) 0 (0.0 %)
**N stage** N0 N1 N2	8 (19.0 %) 8 (19.0 %) 26 (61.9 %)
**M stage** M0/Mx M1	40 (95.2 %) 2 (4.8 %; retroperitoneal lymph node metastasis in 1 patient, hepatic metastasis in 1 patient)
**Grading** G1 G2 G3	0 (0.0 %) 25 (59.5 %) 17 (40.5 %)
**Tumor localization** Pancreatic head Pancreatic body and/ or tail	33 (78.6 %) 9 (21.4 %)
**Type of pancreatectomy** ppWhipple cWhipple Left resection Total pancreatectomy	18 (42.9 %) 9 (21.4 %) 8 (19.0 %) 7 (16.7 %)

Abbreviations: cWhipple, conventional Whipple procedure; IQR, interquartile range; ppWhipple, pylorus-preserving Whipple procedure.

## Abbreviations

5-FU: 5-fluorouracil
^18^FDG: ^18^F-fluorodeoxyglucose
ADC: apparent diffusion coefficient
CI: confidence interval
cWhipple: conventional Whipple procedure
CT: computed tomography
DKFZ: German Cancer Research Center
DW-MRI: diffusion-weighted magnetic resonance imaging
EMT: epithelial-to-mesenchymal transition
GRAPPA: generalized autocalibrating partially parallel acquisitions
HCC: hepatocellular carcinoma
ICC: intra-class correlation coefficient
IQR: interquartile range
MITK: Medical Imaging Interaction Toolkit
MRI: magnetic resonance imaging
PDAC: pancreatic ductal adenocarcinoma
PET: positron emission tomography
ppWhipple: pylorus-preserving Whipple procedure
Runx2: Runt-related transcription factor 2
TGF-β: transforming growth factor-β
VOI: volume of interest
Zeb1: Zinc finger E-box-binding homeobox 1**.**
